# Electrochemical Determination of Norepinephrine by Means of Modified Glassy Carbon Electrodes with Carbon Nanotubes and Magnetic Nanoparticles of Cobalt Ferrite

**DOI:** 10.3390/s18041223

**Published:** 2018-04-16

**Authors:** Daniely Ferreira de Queiroz, Tony Rogério de Lima Dadamos, Sergio Antonio Spinola Machado, Marco Antonio Utrera Martines

**Affiliations:** 1 Laboratório de Materiais Coloidais, Departamento de Físico Química, Instituto de Química, Universidade de São Paulo, Av. Trabalhador São Carlense, C.P. 780, São Carlos 13560-970, SP, Brazil; sasmach@iqsc.usp.br; 2 Instituto de Biociências, Letras e Ciências Exatas, Campus de São José do Rio Preto, Universidade Estadual Paulista, R. Cristóvão Colombo, 2265, Jardim Nazareth, São José do Rio Preto 15054-000, SP, Brazil; tonyrlrl@yahoo.com.br; 3 Laboratório de Química de Superfície e Moléculas Bioativas, Departamento de Química, Universidade Federal do Mato Grosso do Sul, Av. Senador Filinto Müller, 1555, Campo Grande 79074-460, MS, Brazil; marcomartines@gmail.com

**Keywords:** electrode modified, cobalt ferrite, noradrenaline

## Abstract

This study describes the electrochemical preparation of the electrocatalytic oxidation/reduction of noradrenaline in modified glassy carbon of cobalt ferrite nanoparticles and carbon nanotubes (GC/MWCNT/FCo_98_). The cobalt ferrite powder was characterized by X-ray diffraction (XRD) and transmission electron microscopy (TEM). The optimum conditions found in an electrode composition were 4 μL of cobalt ferrite and 10 μL of carbon nanotubes in 0.1 mol L^−1^ PBS at pH 7.0. The electrode displays electrochemical behavior in a wide potential range (−0.4 to 1.0 V vs. Ag/AgCl), high conductivity, and electrode stability/durability in 0.1 mol L^−1^ PBS. Catalytic oxidation of noradrenaline was performed at the unmodified GC electrode at +0.60 V vs. Ag/AgCl and current of 0.17 μA and modified GC with cobalt ferrite nanoparticles and carbon nanotubes at +0.54 V vs. Ag/AgCl and current of 0.23 mA. With regard to the anodic peak current (*I*_pa_) versus noradrenaline concentration by means of the amperometric method at the modified electrode, (which is linear in the 0.16 and 1.91 mmol L^−1^ concentration range), the concentration limit was 0.76 μmol L^−1^. In this way, the modified electrode GC/MWCNT/FCo_98_ was found to be a promising application for the determination of this neurotransmitter in the area of neuroscience.

## 1. Introduction

Nanotechnology is a widespread and multidisciplinary area of research and development, which is based on different types of materials such as polymers, ceramics, and composites. It can be regarded as one of the most exciting innovations in the traditional areas of knowledge [1]. The behavior at the nanoscale as well as its physical and chemical properties is different from that observed at the macroscopic scale [2]. These phenomena, which are caused by the reduction in size to nanoscale dimensions, include confinement resulting from the size of the charge carriers, dependency between electronic structure and particle size, and particles on the interface (surface/volume ratio) [3].

The increased surface area of nanoparticles leads to a significant increase in reactivity, which is useful when conducting a chemical experiment, particularly in the area of biosensors and catalysis. Catecholamines such as dopamine, noradrenaline, and adrenaline play a major role in biological systems. Their importance has led to new research methods for the determination of catecholamines and the control of their activity such as in vivo [4].

Neuroscience is a relatively new field. Research in this area has arisen with the aim of articulating knowledge about the nervous system that is produced independently in different areas. In particular, it is worth highlighting the interaction of neuroscience with a wide range of fields such as chemistry, physics, biology, and several branches within these areas [5]. This interaction has made the study of the nervous system very sophisticated, and brought about new perspectives on the human mind. In neuroscience, attention should be paid to the role of neurotransmitters, including norepinephrine [6]. In the nervous system, neurons can be biochemically differentiated in accordance with their different synaptic neurotransmitter secretions. These are adrenergic, cholinergic, dopaminergic, and serotonergic neurons that secrete norepinephrine, acetylcholine (ach), dopamine, and serotonin, respectively. Thus, the detection of noradrenaline is important for the field of neuroscience [7]. 

Norepinephrine is classified as a catecholamine. Catecholamines play an important role in the central nervous system as neurotransmitters. Accurate and selective measurement of catecholamines such as dopamine, epinephrine, norepinephrine, and serotonin in biological samples is important for clinical diagnosis and the study of certain pathological diseases [8]. In clinical chemistry, the measurement of urinary free catecholamines is widely regarded as a sensitive and suitable screening test to detect brain tumors such as pheochromocytomas and neuroblastomas. This technique also provides additional data that are useful for detecting heart and cardiovascular diseases (e.g., congestive heart failure and hypertension) and diabetes mellitus [9]. Given the transmitter function of catecholamines in the brain, their concentration in body fluids serves as a biochemical indicator of several neurological disorders, including those affecting learning and memory formation. This technique is also useful in other investigations, such as tracing the pathological phases of Parkinson’s disease and assessing the effects of exposure to occupational stress. In view of this, improved methods for detecting catecholamines are needed [10].

Noradrenaline, in particular, is a neurotransmitter of the nervous system and precursor of adrenaline. Norepinephrine has activity in both the adrenergic alpha and beta 1 receptors but has little activity for beta 2 receptors [11]. Depending on the dosage administered, it leads to an increase in stroke volume, as well as being an important peripheral vasoconstriction with an increase in blood pressure. There is also an increase in contractility and cardiac work if the ventricle tolerates the advanced afterload. Norepinephrine is also a potent vasoconstrictor and may impair the function of the kidney, which limits its clinical use [12]. It is also vasoconstrictive within the vascular and pulmonary networks, and should be used with caution in patients suffering from pulmonary hypertension. In the literature several methods are described for the determination of noradrenaline and chemiluminescence [13], mass spectroscopy [14] and other electrochemical techniques [15,16]. This study aims to describe and characterize the electrochemical behavior of noradrenaline in an aqueous solution at the interface of the modified electrode, since these results can be used to improve our understanding of the pharmacodynamics of catecholamines when using a modified glassy carbon electrode with cobalt ferrite nanoparticles and carbon nanotubes.

## 2. Experimental

### 2.1. Reagents and Procedures 

All reactions were carried out through an ultrapure water system and chemical reagents were used as received, without any treatment or purification. The following reagents were employed for the synthesis: cobalt (II) chloride hexahydrate (98% Sigma-Aldrich, St. Louis, MO, USA), iron (III) chloride tetrahydrate (98% Sigma-Aldrich), sodium hydroxide (95% Synth, São Paulo, Brazil), tetraethyl orthosilicate (98% Sigma-Aldrich) and ethanol (98% Sigma-Aldrich).

Carbon nanotubes (MWCNT) (Sigma-Aldrich) and ethanol (Sigma-Aldrich) were used for the surface modification of the glassy carbon electrode. Ten milligrams of MWCNT in 2 mL of ethanol were dispersed for this. The preparation process of the Cobalt ferrite nanoparticles was as described by Silva et al. (2004) [17], in which the hot co-precipitation, which is a dehydration process involving mixed hydroxides and a crystallization of the material required for the formation of CoFe_2_O_4_. After precipitation, it was left for one hour at 98 degrees, forming cobalt ferrite (FCo_98_). The cobalt ferrite nanoparticles (FCo_98_) were formed by dispersing 10 mg FCo_98_ in 2 mL of ethanol. Potassium ferrocyanide (J.T.Baker, Phillipsburg, NJ, USA), potassium ferricyanide (Mallinckrodt, Staines-upon-Thames, UK), and sulfuric acid (Synth) were used for cleaning and characterizing the electrode surface. A solution was formed with 0.01 mol L^−1^ of ferrocyanide/ferricyanide in 0.1 mol L^−1^ of sulfuric acid. Norepinephrine (Fluka, St. Louis, MO, USA) was used as the substrate and sodium phosphate dibasic anhydrous (J.T.Baker) and potassium phosphate monobasic (Merck, Kenilworth, NJ, USA) were used as the supporting electrolytes for the detection of the substrate. The phosphate buffer solution (PBS) was used at a concentration of 0.1 mol L^−1^. All the solutions were prepared by adding purified water to the Millipore Milli-Q system (resistance 18 MWcm).

### 2.2. Instrumentation and Measurement

The materials were characterized by X-ray diffraction (XDR) using a Rigaku^®^ Mini Flex II X-Ray Diffractometer, with Cu K_α_ radiation = 1.54184 Å at 30 kV and 15 mA, in the θ-θ scan mode, and a 2θ angle of 5° to 75° with 0.02° resolution. The surface area was measured through adsorption isotherm of nitrogen at a temperature of 77 K. The morphology of the solid material was investigated by transmission electron microscopy (TEM) using a Philips CM120 microscope operating at 120 kV. The magnetic profile was obtained with the aid of a conventional vibrating sample magnetometer (VSM) with fields of 2 T at room temperature. 

Cyclic voltammetry techniques (CV) were employed for the electrochemical analysis and chronoamperometry was performed by means of a potentiostat/galvanostat Model PGSTAT 30 Autolab electrochemical system (Eco Chemie, Utrecht, The Netherlands) with software coupled to the GPES (Eco Chemie).

Electrochemical measurements were made with the aid of a thermostatic electrochemical cell 15 mL, containing three electrodes: (a) a reference electrode Ag/AgCl/KCl (3.0 mol L^−1^), (b) a platinum electrode as the counter electrode, and (c) a working electrode (area = 0.071 cm^2^). 

Experiments using cyclic voltammetry characterization of the surface of the modified electrode were carried out in a solution of potassium ferrocyanide and potassium ferricyanide 0.01 mol L^−1^ in phosphate buffer 0.1 mol L^−1^ (pH = 7) in the potential range of −0.4 to 1.0 V vs. Ag/AgCl with a scan rate of 0.1 V s^−1^. The cyclic experiments to study the electrochemical behavior of norepinephrine voltammetry were conducted in a phosphate buffer solution 0.1 mol L^−1^ (pH = 7) in the potential range of −0.4 a 1.0 V vs. Ag/AgCl with a scan rate 0.1 V s^−1^. The chronoamperometry for detection of noradrenaline was performed in a phosphate buffer solution 0.1 mol L^−1^ (pH = 7) with a fixed potential 0.5 V vs. Ag/AgCl with a stabilization time of 100 s.

Electrochemical impedance spectroscopy (EIS) was performed using a potentiostat/galvanostat Model PGSTAT 30 Autolab electrochemical system (Eco Chemie) with software coupled to the FRA2 (Eco Chemie). The experiments were performed by means of the EIS in a frequency range of 100 kHz to 40 mHz with an amplitude of 10 mV, and 10 deca points per frequency. The experiments were performed in a solution of potassium ferrocyanide (K_4_Fe(CN)_6_) and potassium ferricyanide (K_3_Fe(CN)_6_) 0.01 mol L^−1^ dissolved in phosphate buffer 0.1 mol L^−1^ (pH = 7).

### 2.3. Preparation of Electrodes

Before modification, the glassy carbon electrode was subjected to polishing with alumina solution (0.05 μm), then rinsed with distilled water and placed in an ultrasound bath for 5 min in ethanol and 5 min in water. After electrochemical polishing, it was dipped in treated with sulfuric acid solution 0.1 mol L^−1^, using cyclic voltammetry in a potential range of −1 to 1 V vs. Ag/AgCl [18]. After this, 10 mg MWCNT and 10 mg FCo_98_ were suspended in 2 mL ethanol. The suspension was dispersed by means of an ultrasound for 30 min. An aliquot of 10 μL of MWCNT and 4 μL of FCo_98_ were dripped onto the surface of the glassy carbon electrode.

### 2.4. Preparation and Analysis of Norepinephrine

The analysis of norepinephrine involved moving an amount equivalent to 10 mmol L^−1^ of norepinephrine (Fluka) that was freshly groomed and dissolved in 25 mL of phosphate buffer 0.1 mol L^−1^ (pH = 7). Aliquots of 10 to 500 µL of noradrenaline were added successively in an electrochemical cell containing 10 mL of phosphate buffer solution 0.1 mol L^−1^ and homogenized with a magnetic stirrer to obtain the chronoamperograms. The chronoamperograms were obtained through a fixed potential 0.5 V vs. Ag/AgCl with a stabilization time of 100 s.

## 3. Results and Discussion

### 3.1. Synthesis and Characterization of Magnetic Particles by a Co-Precipitation CoFe_2_O_4_ Process

**Figure 1:**
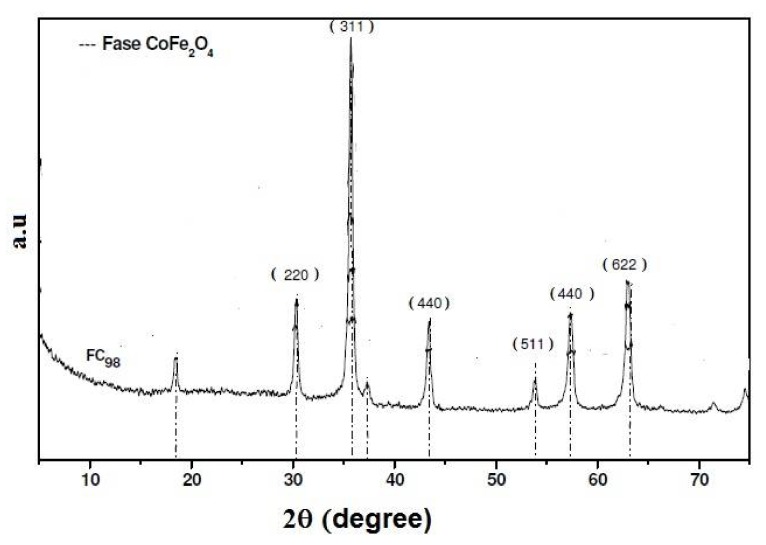
Diffractogram of X-rays of synthesized cobalt ferrites (FCo_98_).

In the formation of ferrite magnetic material, it is necessary to undergo an “aging period,” which is a dehydration process involving mixed hydroxides, and a crystallization of the material required for the formation of CoFe_2_O_4_. The physical properties of the particle, such as growth and phase transformation, may be changed over a period of time as well as the “aging temperature,” due to the growth mechanisms of these particles [17]. The crystalline phases, such as the oxyhydroxide hydroxide, and, in particular the β-FeOOH phase called akaganeite, has a reddish-brown color and magnetic properties of the antiferromagnetic type, and thus the presence of these phases leads to a reduction in magnetic properties [19]. The identification of the crystallographic phase of the synthesized cobalt ferrite (FCo_98_) is shown in the XRD of Figure 1. 

When a comparison was made between the synthesized materials with diffraction patterns (CoFe_2_O_4_, JCPDS 22-1086) and (β-FeOOH, JCPDS 29-0712), the values of the diffraction peaks of 2θ were used where there was the presence of the CoFe_2_O_4_ phase based on the most intense peak in the XRD diffraction index in the plane (311) [20]. The XRD only shows the sample of the FCo_98_ cubic crystalline phase with a peak of greatest intensity in the crystallographic plane (311). The FCo_98_ sample was dried in an oven at a temperature of 100 °C for 2 h, using a mass of 0.1 g of the sample that was analyzed at a temperature of 77 K. The nitrogen adsorption isotherm showed a value of 46 ± 0.14 m^2^/g for the surface area of the ferrite.

**Figure 2:**
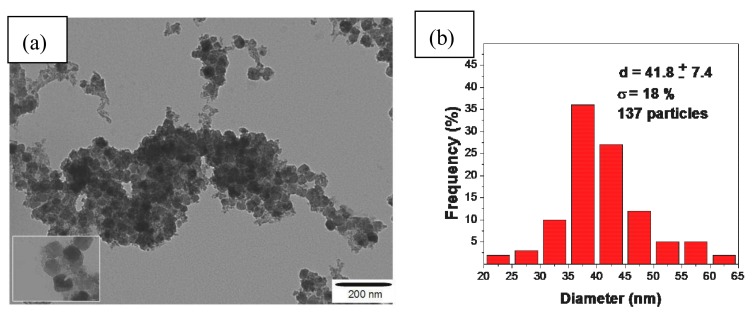
(**a**) Transmission electron microscopy image of the FCo_98_ sample; (**b**) histogram of the amount of particles, depending on the size of the nanoparticles of cobalt ferrite.

Figure 2a shows the TEM image of the FCo_98_ sample, where it was possible to calculate the average diameter of the particle size with the aid of free ImageJ software. The images TEM of glassy carbon electrode and carbon nanotubes have been described in the literature [21,22]. The modification of glassy carbon electrode with carbon nanotubes and FCo_98_ was done by adsorption, not modifying the structures. This obtained an average diameter of 42 nm with a standard deviation of d = ±7.4 nm and polydispersity degree of σ = 18% (as shown in Figure 2b). The degree of polydispersity was calculated by dividing the standard deviation by the mean particle diameter. A monodisperse system is one that has a degree of polydispersity that is less than, or equal to, 10%. On the basis of this criterion, the FCo_98_ sample showed a wide particle size range that confirmed the degree of polydispersity, (as can be seen in Figure 2b).

### 3.2. Electrochemical Behavior GC/MWCNT/FCo_98_

**Figure 3:**
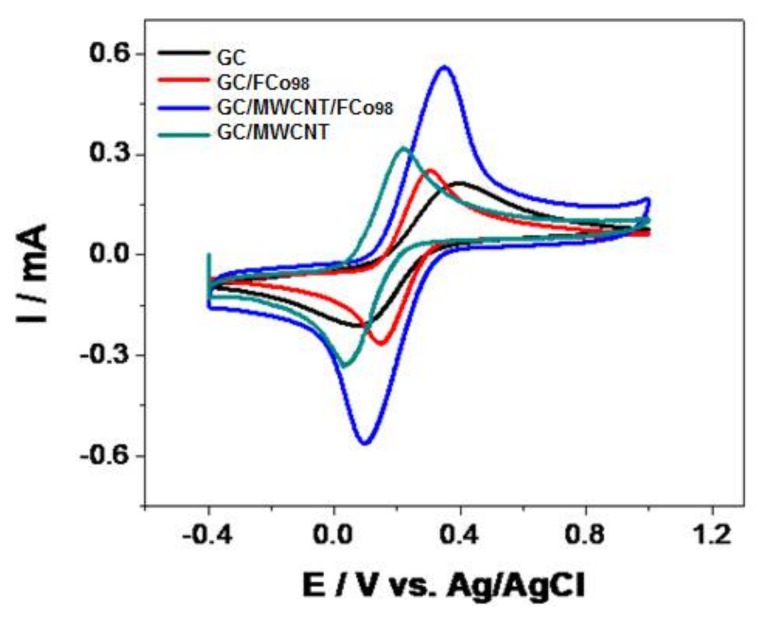
Cyclic voltammograms for potassium ferrocyanide/potassium ferricyanide 0.01 mol L^−1^ in phosphate buffer 0.1 mol L^−1^ (pH = 7) with a scan rate of 100 mV s^−1^ of GC (───), GC/ FCo_98_ (───), GC/MWCNT (───), GC/MWCNT/FCo_98_ (───).

The study of the modification of glassy carbon electrodes with nanoparticles of cobalt ferrite and carbon nanotubes was carried out by means of cyclic voltammetry in the potential range of −0.4 to 1.0 V vs. Ag/AgCl with a scan rate of 100 mV s^−1^ using an electron mediator redox couple, and potassium ferrocyanide/potassium ferricyanide 0.01 mol L^−1^ in phosphate buffer 0.1 mol L^−1^ (pH = 7). Figure 3 shows the cyclic voltammograms.

**Figure 4:**
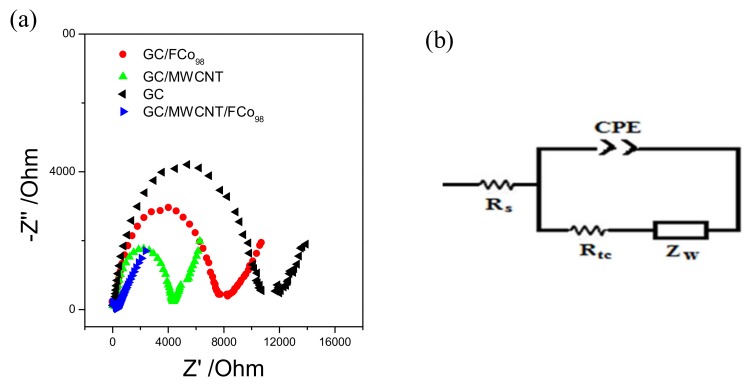
(**a**) Complex plane spectra obtained for the modified electrode with GC (─), GC/ FCo_98_ (─), GC/MWCNT (─) and GC/MWCNT/FCo_98_ (─) using a 100 kHz frequency band at 0.1 Hz, 10 mV sine wave applied at the *E*pa = 0.4 V vs. Ag/AgCl. The lines that go beyond the identifiers are the simulation results with software that employ the modified Randles circuit. (**b**) An equivalent circuit model was used to analyze the data, together with the complex plane graphs obtained from the EIS.

As seen in Figure 3, voltammograms of the modified electrode with nanoparticles of cobalt ferrite and carbon nanotubes, have a more clearly defined voltammetric profile with a decrease of the 100 mV overpotential vs. Ag/AgCl and an increase of current by a factor of 2.6 times. The electrochemical impedance spectroscopy technique (as shown in Figure 4), was used to characterize the modified electrode with cobalt ferrite nanoparticles and carbon nanotubes.

We used a solution of potassium ferricyanide and potassium ferrocyanide in 0.1 mol L^−1^ PBS. A sine wave was applied with 10 Mv pulse amplitude of the peak potential, which during the cyclic voltammetry experiments was observed in a frequency range of 100 kHz to 0.1 Hz. The circuit model were used for the spectra was Randles *R_s_* (CPE[R_ct_Z_w_]). The data obtained are shown in Table 1. 

The data in Table 1 show that the values of resistance and capacitance vary for each electrode studied and thus demonstrate the effects of modifying the electrode with the cobalt ferrite and carbon nanotubes. The use of low-amplitude sine wave allowed the frequency regions to be clearly separated where the redox pair [Fe(CN)_6_]^4−^/[Fe(CN)_6_]^3−^ occurs by is under kinetic control and control of mass transport. Under these conditions, the rate constant of heterogeneous electron transfer is apparent (*K_app_*) and the reaction can be expressed by Equation (1) [23]:

(1) $$K_{app}=\frac{RT}{F^{2}R_{tc}CA},$$

where *F* is the Faraday constant (96,485 C mol^−1^), *C* concentration [Fe(CN)_6_]^4−/3−^ in solution (0.01 mol L^−1^), *R* is the gas constant physical value (8.3145 J K mol), *T* the temperature in Kelvin (298 K), *A* is area of the electrode (0.071 cm^2^) and *R_tc_* the cargo transfer of resistance. Values *K_app_* are shown in Table 1. According to the values of *K_app_*, it can be seen that the electrode GC/MWCNT/FCo_98_ has a value 87 times higher than for the GC, 22 times greater for the GC/MWCNT and 44 times greater for the GC/FCo_98_, which demonstrates that electron transfer is much faster by means of the modified electrode with cobalt ferrite and a carbon nanotube.

As a means of investigating the properties of FCo_98_ nanoparticles on the glassy carbon substrate, a study was carried out of the influence of the amount of nanoparticles on the electrode surface using ferricyanide/ferrocyanide in 0.1 mol L^−1^ PBS. These were deposited on the surface of 1 to 10 μL nanoparticles, starting with a solution of 10 mg of nanoparticles dispersed in 2 mL of ethanol. A potential range was used of −0.4 to 1.0 V vs. Ag/AgCl with a scan rate of 100 mV s^−1^. 

It was noted that the 1 to 4 μL of FCo_98_ on the electrode surface causes an increase in the current magnitude of the redox peak. This behavior shows that when the amount of FCo_98_ increases to 4 μL, it leads to an increase in the surface area, which facilitates the electron transfer across the electrode surface [24]. However, amounts greater than 4 μL decrease the magnitude of the redox peak current, which is due to the isolation of the electrode surface because of the large amount of nanoparticles covering the electrode [25].

### 3.3. Detection of Norepinephrine with a Modified GC/MWCNT/FCo_98_ Electrode

**Figure 5:**
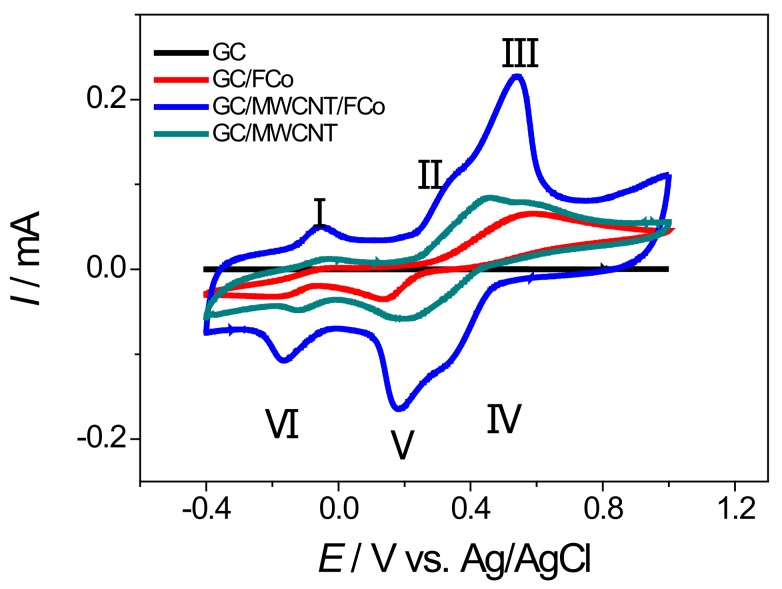
Cyclic voltammograms in the presence of noradrenaline 10 mmol L^−1^ in phosphate buffer 0.1 mol L^−1^ (pH = 7) with a scan rate of 100 mV s^−1^ of GC (───), GC/ FCo_98_ (───), GC/MWCNT (───), GC/MWCNT/FCo_98_ (───).

Experiments were carried out with the aim of clarifying the processes of oxidation and reducing the noradrenaline on the modified glassy carbon electrode with nanoparticles of cobalt ferrite and carbon nanotubes. These made use of cyclic voltammetry in the potential range of −0.4 to 1.0 V vs. Ag/AgCl with a scan rate of 100 mV s^−1^ using a solution of 10 mmol L^−1^ of norepinephrine in phosphate buffer 0.1 mol L^−1^ (pH = 7). Figure 5 shows the cyclic voltammograms containing four types of electrodes: GC, GC/ FCo_98_, GC/MWCNT and GC/MWCNT/FCo_98_. 

The voltammograms show that in the potential window studied, there are three redox processes of oxidation/reduction to the electrode GCMWCNT/ FCo_98_. The cyclic voltammograms show a redox couple with an *E*_pa_ at about +0.34 and 0.54 V (Peak II and III, respectively) and an *E*_pc_ at about +0.35 and 0.19 V vs. Ag/AgCl (peak IV and V, respectively). As predicted, an oxidation peak at +0.34 and 0.54 V was observed in the positive scan, which can be attributed to the oxidation of noradrenaline (transfer of 2e^−^ and 2H^+^). Another peak was observed in the subsequent reverse segment at +0.35 and 0.19 V, which can be attributed to a reduction of the *o*-quinone form of noradrenaline, with a peak-to-peak separation of about 190 mV (peak II and V) and 190 mV (peak III and IV) [26]. Equation (2) shows this process.

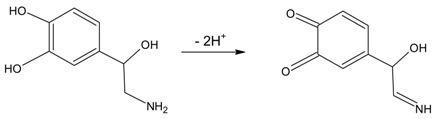
(2)

According to Luczac (2007) [27], the redox process for Peaks I (*E*_pa_ −0.05 V) and VI (*E*_pc_ −0.17 V) relates to the oxidation and reduction of amino groups present in the molecule of norepinephrine, with a peak-to-peak separation of about 120 mV. Equation (3) shows this process.

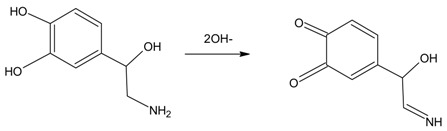
(3)

As observed in the voltammograms of Figure 5, a modified electrode with cobalt ferrite nanoparticles and carbon nanotubes has a more clearly defined voltammetric profile with a decrease of the overpotential 60 mV vs. Ag/AgCl. In Figure 5, there is an increase of current by a factor of 1300 times compared with the GC electrode; 3.4 times compared with the GC/FCo_98_ electrode and 2.7 times compared with the GC/MWCNT electrode. This demonstrates that the modified GCMWCNT/FCo_98_ electrode has a lower oxidation potential for noradrenaline, which prevents the interference of oxidation, and a higher peak current for oxidation of noradrenaline and thus shows a higher sensitivity in the detection of the analytes. The repeatability of the voltammetric measurements was evaluated by recording 10 successive cyclic voltammograms of the modified glassy carbon electrode in PBS 0.1 mol L^−1^ solution (pH 7.0) containing noradrenaline, without renewing the electrode surface between successive runs. The anodic peak current was measured in accordance with the set of optimum conditions described in Table 2.

The ratio of cathodic to anodic peak currents at various square root scan rates was almost uniform. The electrode reduction and oxidation peak currents for entrapped noradrenaline are found to increase linearly with square root scan rates ranging from 20 to 500 mV s^−1^. The linear correlation of the peak current with a square root scan rate, showed that the system is similar to the process controlled by the diffusion mechanism [28]. The linear relationship for anode was straight I (μA) = 2.94 + 7.0 v^1/2^ (mV s^−1^) and the cathode line was I (μA) = 7.14 − 6.08 v^1/2^ (mV s^−1^). The linearity of the logarithm of the peak current (log *I*_p_) vs. the logarithm of scan rate (log v), was obtained by means of slope coefficients from 0.46 to the anodic process, and the 0.57 process for the cathode. The values obtained are similar to the theoretical value of 0.5 for diffusion processes [28]. It can be concluded from these results that the charge transfer reaction of noradrenaline is controlled by diffusion of noradrenaline in the solution for surface electrodes.

The apparent electrochemical rate constant *k*_e_ was calculated for the noradrenaline by employing the method described by Laviron [29]. It was shown by Laviron that f, *k*_e_ can be determined for a surface redox couple from the variation of *E*_pa_ and *E*_pc_ with a suitable can rate. When the scan rates were large enough, the *E*_p_ vs. log v plots gave two straight lines with slopes for the cathodic branch and for the anodic branch. The apparent electrochemical rate constant can then be determined by applying the equation *k*_e_ = 2.303α_an_Fv_o_/RT, where R is the gas constant, T the absolute temperature, F the Faraday constant, and α an anodic transfer coefficient involved in the redox process. When calculating the anodic transfer coefficient, (α) was applied to the relation α = 2.303RT/nFθ, where θ is the inclination angle for the anode. The value of the scan rate (v_o_) is determined by extrapolating the linear branch at higher scan rates and at its intersection with the constant peak potential, represented by the peak of the voltammogram at the lower scan rate. The observed value was *k*_e_ = 7.99 s^−1^; this value is similar to those reported for well-known moderate mediators [29]. 

The surface concentration of species on electrode surfaces (Γ_GCMWCNT/FCo98_/mol cm^−2^) was estimated from the background-corrected electric charge, Q, under the anodic peaks in accordance with the theoretical relationship [30] (expressed in Equation (4)).

(4) $$\Gamma =\frac{Q}{nFA},$$

where *n* represents number of electrons transferred (assume ≈ 2), F the Faraday constant and A is the geometric surface area of the electrode (0.071 cm^2^). After cycling the electrode cells in 10 mmol L^−1^ noradrenaline and in 0.1 mol L^−1^ PBS at 100 mV s^−1^, the estimated surface concentration of the noradrenaline was found to equal 2.49 × 10^−8^ mol cm^−2^.

The effect of pH on the formal potential and anodic peak current was investigated by cyclic voltammetry in the solution containing noradrenaline (the pH of the PBS buffer was varied adjusted by adding HCl or NaOH). The values of the formal potential and anodic peak current were dependent on the pH of the buffer solution, which shows that the redox couple of the noradrenaline includes some proton transfer in the reduction and oxidation processes. The *I*_p_ vs. pH plot reveals a maximum current at approximately pH 7.0, which decreases again at a higher pH. 

### 3.4. Amperometric Measurement of Noradrenaline

**Figure 6:**
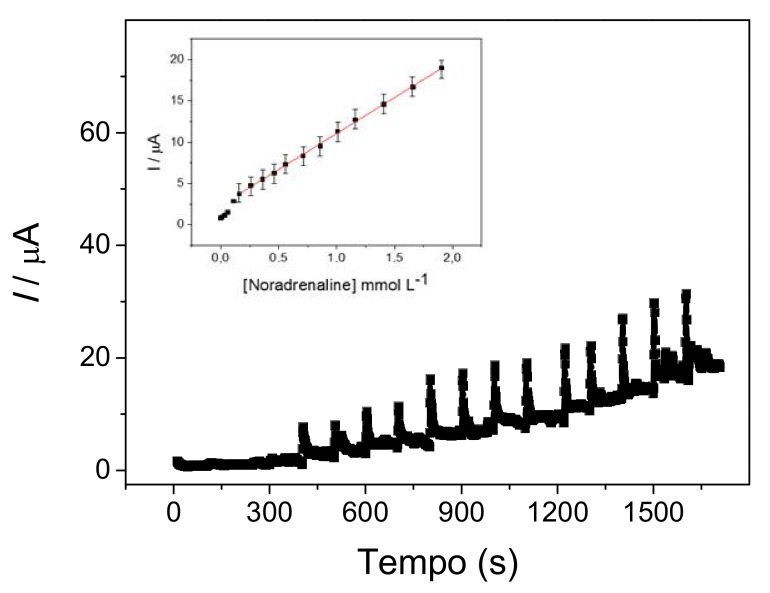
Calibration Curve based on the chronoamperometric response of a modified electrode with a GC/MWCNT/FCo_98_ in PBS 0.1 mol L^−1^ solution (pH 7.0) for 12 additions of 10 mmol L^−1^ noradrenaline at a constant potential rate of 0.5 V vs. Ag/AgCl.

An attempt was made to evaluate the performance of the GC/MWCNT/FCo_98_ as an amperometric sensor for noradrenaline in PBS 0.1 mol L^−1^. Experiments of chronoamperometry were conducted at different applied potentials (0.1–0.7 V vs. Ag/AgCl) in a modified electrode to find the best potential system for the amperometric determination of noradrenaline. However, since the stirring of the solution was slow, the loss of the formed film did not occur [31]. The highest amperometric response to noradrenaline was obtained at 0.5 V vs. Ag/AgCl. An increase in amperometric response was observed in the more positive potential values, probably due to the noradrenaline adsorption on the electrode surface. A standard calibration curve (Figure 6) with statistical data was obtained by the successive addition of amounts of noradrenaline to 10 mL of 0.1 mol L^−1^ PBS by means of the cronoamperometry method, with a potential of 0.5 V vs. Ag/AgCl. 

The electrode response time was fast for different noradrenaline concentrations and showed stable currents in a few seconds (100 s). In this curve, the anodic peak current at the GC/MWCNT/FCo_98_, was in a range of 0.16 to 1.91 mmol L^−1^ with a detection limit (Miller & Miller) [32] of 0.76 μmol L^−1^. The linear regression equation is: *I*_pa_(A) = 2.34 × 10^−6^ ± 0.03 × 10^−6^ (A/L mol^−1^) + 0.82 × 10^−3^ ± 0.03 × 10^−3^ [noradrenaline] (mol L^−1^); *r* = 0.9989; *n* = 12 with residual 0.052 × 10^−6^. According to Goldstein et al., (1986) [33] the concentration variation of noradrenalide concentration in neural fluids, ranges from 3 to 21 nmol/L, where the LOD value found was close to that expected for this type of determination. This limit can be exceeded by using pulsed techniques (differential pulse voltammetry and square wave voltammetry) that are sensitive rather than amperometric.

## 4. Conclusions

The GC/MWCNT/FCo_98_ showed an electrocatalytic effect in the oxidation/reduction of noradrenaline. The most significant factor observed in this work is the electrocatalytic activity of the material in the noradrenaline oxidation/reduction at a relatively low applied potential. The optimum conditions for the analysis of noradrenaline were as follows: 4 μL FCo_98_ and 10 μL MWCNT, and a scan rate of 100 mV s^−1^ between −0.4 and 1.0 V vs. Ag/AgCl. The anodic peak current increased significantly and anodic overpotential was reduced by +0.06 V and an increase in current that was 1330 times more than that obtained at a non-modified electrode in a solution containing noradrenaline. This anodic current was proportional to the concentration of noradrenaline in the range of 0.16 to 1.91 mmol L^−1^; the concentration limit was 0.76 μmol L^−1^. The oxidation/reduction of noradrenaline on the sensor seems to follow a catalytic reaction mechanism, as described in Equations (2) and (3). In view of its good stability, the modified electrode shows promise as a means of developing a new sensor for noradrenaline detection in neuroscience, together with a voltammetric and amperometric analysis. In future work, a more detailed study of the electroactive surface of the electrode modified using the techniques of Atomic Force Microscopy and Transmission Electron Microscopy will necessary for better understanding of the roughness and the arrangement of cobalt ferrite nanoparticles and nanotube carbon on the electrode surface

## Figures and Tables

**Table 1:** Data obtained through the spectra of the complex plane.

Electrode	*R_s_* (Ω)	CPE (µF)	*R_tc_* (Ω)	α	*K_app_* (µcm s^−1^)
GC/FCo_98_	28	3.31	4892	0.91	0.08
GC	29	1.17	10,678	0.98	0.04
GC/MWCNT	31	1.9	2322	0.95	0.16
GC/MWCNT/FCo_98_	31	230	107	0.92	3.5

**Table 2:** Optimized cyclic voltammetry parameters for noradrenaline determination using the modified glassy carbon electrode with cobalt ferrite and carbon nanotubes.

Parameter	Optimum Value
Composition	4 uL FCo_98_ and 10 uL MWCNT
Supporting electrolyte and pH	0.1 mol L^−1^ PBS (pH 7.0)
Potential range and Scan rate	Scan rate of 100 mV s^−1^ between −0.4 and 1.0 V vs. Ag/AgCl.

## References

[1] Vafafard A., Goharshenasan S., Nozari N., Mortezapour A., Mahmoudi M. (2013). Phase-Dependent Optical Bistability in the Quantum Dot Nanostructure Molecules via Inter-Dot Tunneling. J. Lumin..

[2] Rangasamy M. (2011). Nano Technology: A Review. J. Appl. Pharm. Sci..

[3] El-Sayed M.A. (2001). Some Interesting Properties of Metals Confined in Time and Nanometer Space of Different Shapes. Acc. Chem. Res..

[4] Perry M., Li Q., Kennedy R.T. (2009). Review of Recent Advances in Analytical Techniques for the Determination of Neurotransmitters. Anal. Chim. Acta.

[5] Kozikowski A.P. (2011). On the Path from Chemistry to Neuroscience: Early Explorations in Chemical Medicine under the Mentorship of Dr. Erminio Costa, a Neuroscientist with a Big Brain and a Bigger Heart. Pharmacol. Res..

[6] Liu J., Liu Y., Xu Y., Wang M., Zhu Q., Li W., Wang X., Liu J., Ma B., Wu K. (2013). Neurotransmitter Noradrenaline Downregulate Cytoskeletal Protein Expression of Vsmcs. Exp. Mol. Pathol..

[7] Taei M., Ramazani G. (2014). Simultaneous determination of norepinephrine, acetaminophen and tyrosine by differential pulse voltammetry using Au-nanoparticles/poly(2-amino-2-hydroxymethyl-propane-1,3-diol) film modified glassy carbon electrode. Colloids Surf. B Biointerfaces.

[8] Wang Z., Wang K., Zhao L., Chai S., Zhang J., Zhang X., Zou Q. (2017). A novel sensor made of Antimony Doped Tin Oxide-silica composite sol on a glassy carbon electrode modified by single-walled carbon nanotubes for detection of norepinephrine. Mater. Sci. Eng. C.

[9] Mahdavi M.R.V., Roghani M., Baluchnejadmojarad T. (2011). The Role of Adrenergic and Angiotensinergic Systems in Vascular Effect of Alcoholic of Extract Trigonella Foenum-Graecum Seed in Diabetic Rats. Iran. J. Pharm. Res..

[10] Xie Y.C., Huang H.W., Zhang Q.M., Jin S.H. (2009). Quantitative Determination of Adrenaline Hydrochloride Injection and Noradrenaline Bitartrate Injection by a New Hplc Method Via Substitute for Reference Substance. Chem. Res. Chin. Univ..

[11] Gutiérrez A., Primo E.N., Eguílaz M., Parrado C., Rubianes M.D., Rivas G.A. (2017). Quantification of neurotransmitters and metabolically related compounds at glassy carbon electrodes modified with bamboo-like carbon nanotubes dispersed in double stranded DNA. Microchem. J..

[12] Lavanya N., Sekar C. (2017). Electrochemical sensor for simultaneous determination of epinephrine and norepinephrine based on cetyltrimethylammonium bromide assisted SnO_2_ nanoparticles. J. Electroanal. Chem..

[13] Chen F.N., Zhang Y.X., Zhang Z.J. (2007). Simultaneous Determination of Epinephrine, Noradrenaline and Dopamine in Human Serum Samples by High Performance Liquid Chromatography with Chemiluminescence Detection. Chin. J. Chem..

[14] Thomas A., Geyer H., Mester H.J., Schänzer W., Zimmermann E., Thevis M. (2006). Quantitative Determination of Adrenaline and Noradrenaline in Urine Using Liquid Chromatography-Tandem Mass Spectrometry. Chromatographia.

[15] Amiri-Aref M., Raoof J.B., Ojani R. (2014). A highly sensitive electrochemical sensor for simultaneous voltammetric determination of noradrenaline, acetaminophen, xanthine and caffeine based on a flavonoid nanostructured modified glassy carbon electrode. Sens. Actuators B Chem..

[16] Pahlavan A., Gupta V.K., Sanati A.L., Karimi F., Yoosefian M., Ghadami M. (2014). ZnO/CNTs nanocomposite/ionic liquid carbon paste electrode for determination of noradrenaline in human samples. Electrochim. Acta.

[17] Silva J.B., Brito W., Mohallem N.D.S. (2004). Influence of heat tretment on cobalt ferrit powders. Mater. Sci. Eng. B.

[18] Da Silva H., Pacheco J.G., Magalhães J.M., Viswanathan S., Delerue-Matos C. (2014). Mip-Graphene-Modified Glassy Carbon Electrode for the Determination of Trimethoprim. Biosens. Bioelectron..

[19] Rejandra M., Pullar R.C., Bhattacharya A.K., Das D., Chintalapudi S.N., Majumdar K.C. (2001). Magnetic properties of nanocrystalline CoFe_2_O_4_ powders prepared at room temperature: Variation with crystallite size. J. Magn. Magn. Mater..

[20] Li S., John V.T., O’Connor C., Harris V., Carpenter E. (2000). Cobalt ferrite nanoparticles: Struture, cation distributions and magnetic properties. J. Appl. Phys..

[21] Yi Y., Weinberg G., Prenzel M., Greiner M., Heumann S., Becker S., Schlögl R. (2017). Electrochemical corrosion of a glassy carbon electrode. Catal. Today.

[22] Fang W., Linder M.B., Laaksonen P. (2018). Modification of carbon nanotubes by amphiphilic glycosylated proteins. ‎J. Colloid Interface Sci..

[23] Sabatani E., Rubinstein I., Maoz R., Sagiv J. (1987). Organized Self-Assembling Monolayers on Electrodes. Part I. Octadecyl Derivatives on Gold. J. Electroanal. Chem..

[24] Kim S., Na J., Lee S.K., Song M.J., Kang P., Huh J., Lim D.S., Kim G.T. (2013). Geometrical Effects of Nanowire Electrodes for Amperometric Enzyme Biosensors. Sens. Actuators B Chem..

[25] Kikandi S.N., Okello V.A., Wang Q., Sadik O.A., Varner K.E., Burns S.A. (2011). Size-Exclusive Nanosensor for Quantitative Analysis of Fullerene C 60. Environ. Sci. Technol..

[26] Moraes F.C., Cesarino I., Coelho D., Pedrosa V.A., Machado S.A.S. (2012). Highly Sensitive Neurotransmitters Analysis at Platinum-Ultramicroelectrodes Arrays. Electroanalysis.

[27] Łuczak T. (2007). Structure—Reactivity Relationships: The Oxidation of Aliphatic Amines on the Gold Electrode. J. Appl. Electrochem..

[28] Chen L., Lu G. (2006). Direct Electrochemistry and Electrocatalysis of Hybrid Film Assembled by Polyelectrolyte-Surfactant Polymer, Carbon Nanotubes and Hemoglobin. J. Electroanal. Chem..

[29] Laviron E. (1974). Adsorption, Autoinhibition and Autocatalysis in Polarography and in Linear Potential Sweep Voltammetry. J. Electroanal. Chem..

[30] Liu S.Q., Ju H.X. (2002). Renewable Reagentless Hydrogen Peroxide Sensor Based on Direct Electron Transfer of Horseradish Peroxidase Immobilized on Colloidal Gold-Modified Electrode. Anal. Biochem..

[31] Cosio M.S., Pellicanò A., Brunetti B., Fuenmayor C.A. (2017). A simple hydroxylated multi-walled carbon nanotubes modified glassy carbon electrode for rapid amperometric detection of bisphenol A. Sens. Actuators B Chem..

[32] Miller J.N., Miller J.C. (2005). Statistics and Chemometrics for Analytical Chemistry.

[33] Goldstein D.S., Zimlichman R., Stull R., Keiser H.R., Kopin I.J. (1986). Estimation of intrasynaptic norepinephrine concentrations in humans. Hypertension.

